# Quality attributes of sweet potato flour as influenced by variety, pretreatment and drying method

**DOI:** 10.1002/fsn3.325

**Published:** 2015-12-23

**Authors:** Ganiyat O. Olatunde, Folake O. Henshaw, Michael A. Idowu, Keith Tomlins

**Affiliations:** ^1^ Department of Food Science and TechnologyFederal University of AgricultureAbeokutaOgun StateNigeria; ^2^ Foods and Markets DepartmentNatural Resources InstituteUniversity of GreenwichMedwayKentUnited Kingdom

**Keywords:** Drying, food quality, pretreatment, sweet potato flour, variety

## Abstract

The effect of pretreatment methods (soaking in water, potassium metabisulphite solution, and blanching) and drying methods (sun and oven) on some quality attributes of flour from ten varieties of sweet potato roots were investigated. The quality attributes determined were chemical composition and functional properties. Data obtained were subjected to descriptive statistics, multivariate analysis of variance, and Pearson's correlation. The range of values for properties of sweet potato flour were: moisture (8.06–12.86 ± 1.13%), starch (55.76–83.65 ± 6.82%), amylose (10.06–21.26 ± 3.92%), total sugar (22.39–125.46 ± 24.68 *μ*g/mg), water absorption capacity (140–280 ± 26), water solubility (6.89–26.18 ± 3.80), swelling power (1.66–5.00 ± 0.50), peak viscosity (24.50–260.92 ± 52.61 RVU), trough (7.08–145.83 ± 34.48 RVU), breakdown viscosity (11.00–125.33 RVU), final viscosity (10.21–225.50 ± 60.55 RVU), setback viscosity (3.04–92.21 RVU), peak time (6.07–9.06 min) and pasting temperature (69.8–81.3°C). Variety had a significant (*P* < 0.001) effect on all the attributes of sweet potato flour. Pretreatment did not significantly (*P* > 0.05) affect moisture, fat and lightness (L*). Drying method did not significantly (*P* > 0.05) affect fiber and L*. The interactive effect of variety, pretreatment and drying method had a significant (*P* < 0.001) effect on all the attributes except fat and fiber. Total sugar correlated significantly (*P* < 0.01) with water solubility (*r* = 0.88) of the flour samples. Variety was a dominant factor influencing attributes of sweet potato flour and so should be targeted at specific end uses.

## Introduction

Sweet potato [*Ipomoea batatas* L. (Lam.)] is among the world's most important, versatile and underexploited food crops. Nigeria is the leading producer of sweet potato (SP) in Africa with an estimated average production (1993–2013) of 3.45 million metric tonnes (FAOSTAT, 2013). A large number of SP varieties exist and they differ from one another in the color of flesh, and root skin amongst other attributes (Woolfe 1992; Aina et al. 2009). In Nigeria, the two common local varieties are the purple skin–white fleshed and the yellow skin‐yellow fleshed. However, improved varieties including orange‐fleshed varieties, with varying genetic and agronomic characteristics are been developed in Nigerian research institutions and released to farmers (Afuape 2009, 2013; Egeonu and Akoroda 2009).

SP has been recognised as having an important role to play in improving household and national food security, health, and livelihoods of poor families in sub‐Saharan Africa (CIP, 2013). This may be due to its wide range of agronomic and nutritional advantages such as high yield even in marginal soil conditions, wide ecological adaptability, low input requirements, and shorter growing period than other root crops (Horton et al. 1989). SP produces the highest amount of edible energy per hectare per day (Horton et al. 1989). Despite its high carbohydrate content, it has a low glycemic index, indicating low digestibility of the starch (ILSI, 2008). It is the only starchy staple, which contains appreciable amounts of *β*‐carotene (especially the orange‐fleshed varieties), ascorbic acid and amino acid lysine that is deficient in cereal‐based diets like rice (Bradbury and Singh, 1986; Bradbury et al., 1985).

Processing of SP roots into stable forms such as chips, flour, or starch, have been recommended as an alternative to the difficulties associated with storage and transportation of the raw roots in developing countries (Peters and Wheatley, 1997; Owori and Agona 2003). Flour produced from SP has the potential for making a variety of food products such as baked goods (bread, cakes, cookies, biscuits); doughnuts, breakfast foods (instant porridge, crisp, flake‐type products); noodles or pasta‐type products; sauces (soy sauce, ketchup); and brewing adjuncts (van Hal 2000; Mais and Brennan 2008).

Functional quality of flour is important to determine its usefulness in food applications. Processing conditions have been shown to influence functional properties of flour; for example, heat processing have been found to affect the functional properties of taro flour (Tagodoe and Nip 1994), while drying temperature, milling procedure and particle size influenced gelatinization profiles of cassava flour (Fernandez et al. 1996). Factors that have been reported to influence the quality of SP flour are variety (Osundahunsi et al. 2003; Aina et al. 2009), processing steps (van Hal 2000), as well as processing methods such as parboiling (Osundahunsi et al. 2003), blanching (Jangchud et al. 2003), drying techniques (Yadav et al. 2006) and peeling, pretreatments and drying temperatures (Maruf et al. 2010a,b).

Most technical research on SP flour has focused on the development of new food products using SP flour rather than on efficient methods to produce the flour (van Hal 2000). Meanwhile, researchers have reported different characteristics of SP flour processed from different varieties and under different conditions (van Hal 2000; Jangchud et al. 2003; Osundahunsi et al. 2003; Yadav et al. 2006; Maruf et al.*,* 2010a, b). There is need however to harmonize and apply some common processing conditions of pretreatment and drying that has been studied, and report the quality of flour in relation to several varieties. This is important in order to understand the effect of interactions among these independent variables on quality attributes of SP flour. This study was conducted to evaluate the effect of variety, pretreatment, and drying methods on chemical composition and functional properties of SP flour. The functional characterization of flour from varieties of SP roots used in this study was also conducted.

## Methodology

### Sweet potato roots

Ten varieties of mature sweet potato roots (Table 1) were used for this study. Sweet potato vines were transplanted into the main field in June/July and mature roots were harvested in 4 months after planting in October/November.

**Table 1:** Characteristics of sweet potato roots

Variety	Status	Color of flesh
LWF^a^	Local	White
LYF^b^	Local	Yellow
Arrowtip	Improved	White
TIS 87/0087	Improved	White
TIS 2531 OP‐1‐13	Improved	White
TIS 8250	Improved	White
Shaba	Improved	Yellow
CIP Tanzania	Improved	Yellow
199034.1	Improved	Orange
Ex‐Oyunga	Improved	Orange

a LWF (local white‐fleshed) referred to as “Anama funfun” in local Yoruba dialect.

b LYF (local yellow‐fleshed) referred to as “Anama funfun” in local Yoruba dialect.

### Sweet potato flour

Sweet potato roots were washed with tap water to remove soil particles. The cleaned roots were air‐dried, peeled manually with stainless steel kitchen knife and sliced into 2 mm thickness (Jangchud et al. 2003), using a domestic plantain slicer. Each variety was subjected to four different pretreatment conditions (1) soaked in water for 90 min (Owori and Hagenimana 2000), (2) soaked in 0.5% potassium metabisulphite solution for 30 min followed by soaking in water for 45 min (Oduro et al. 2003), (3) blanched at 70°C for 5 min (modified from Jangchud et al. 2003), and (4) no pretreatment (control). Each pretreated sample was subjected to each of two drying methods; (i) sun for 3–4 days and (ii) oven at 50°C for 5 h (Woolfe 1992; van Hal 2000). The dried SP chips were stored in heat‐sealed polyethylene bags at room temperature (30 ± 2°C) and transported to the laboratory of the Natural Resources Institute, University of Greenwich, United Kingdom. The chips were initially reduced into smaller particles in a blender (VORWERK Thermomix 31‐1, France), followed by milling into flour using a laboratory mill (Perten 3600) and sieving through a 250 *μ* aperture screen (Endecotts, London, UK.). The flour samples were packed in resealable polyethylene bags and stored at −10 ± 3°C prior to analysis.

### Flour analyses

#### Proximate composition

Standard methods were used to determine moisture, protein, fat, fiber (AOAC, 2000), and ash (AOAC, 1984). Total carbohydrate was determined by difference (James 1995) as: Total carbohydrate (%) = 100 – (% moisture + % protein + % fat + % fiber + % ash).

#### Chemical properties of flour

Starch content was determined using the Phenol‐sulphuric acid method (Dubois et al. 1956).

A quantity of 50 g of flour was extracted with hot 80% ethanol to separate the sugar. 1.0 mL of the sugar extract was pipetted into a test tube and diluted to 2.0 mL with distilled water. 1.0 mL of 5% phenol was added and mixed thoroughly. 5.0 mL of concentrated sulphuric acid was added and the tube was allowed to stand for 10 min. The mixture was vortexed and allowed to stay for another 20 min. Absorbance was read at 490 nm. A standard curve was plotted using 0–100 *μ*g glucose. A standard solution of glucose was prepared by dissolving 10 mg of glucose in 100 mL distilled water. 0.20, 0.40, 0.60, 0.80, and 1.00 mL of the standard glucose solution was pipetted into a test tube and treated following the procedure for sugar extract. The amount of sugar in the flour sample was determined by reference to the standard curve, while taking the dilution factor and weight of sample into consideration.

$$\begin{array}{c} \text{Starch was calculated using this formula:}\\ \mathrm{Starch}(\%)=\frac{0.05\;\times \;A\;\times \;1\;/M}{\text{Weight of sample}}\times 0.9 \end{array}$$

where *A *= Absorbance, *M *= Slope of standard curve.

Amylose content was estimated by the rapid colorimetric method (Williams et al.^1970^). Here 20 mg of flour was weighed into a 50 mL beaker and 10 mL of 0.5 N KOH solution was added. The mixture was stirred with a stirring rod until the flour was fully dispersed in the solution. The dispersed mixture was transferred into a 100 mL volumetric flask and diluted to the mark with distilled water. 10 mL of the diluted test solution was pipetted into a 50 mL volumetric flask, 5 mL of 0.1 N HCl was added, followed by 0.5 mL of iodine reagent. The volume was diluted to 50 mL and the absorbance was measured at 625 nm after 5 min. Amylose was calculated as follows: Amylose (%) = (85.24 × *A*)–13.19 where *A *= Absorbance.

Sugar content was determined by a reversed‐phase high performance liquid chromatography as described by Picha (1985) by extraction of sugars from the samples of flour; values were expressed as *μ*g/mg of fructose, glucose, sucrose and total sugar. Sugar was extracted from 0.2 g of flour sample using 100 mL of 80% ethanol in 2 mL eppendorf tubes. Each tube was vortexed and placed in a water bath at 70°C with agitation for 2 h. The tubes were vortexed again after 30, 60 and 90 min. The tubes were then centrifuged for 4 min at 10,000 rpm (7,826 g) in a microcentrifuge. Thereafter, the extract was filtered into glass High Performance Liquid Chromatography (HPLC) vials using special syringe filters. The sugars were separated through Agilent Zorbax Carbohydrate column (150 mm × 4.6 mm × 5 *μ*m), Agilent Zorbax NH_2_ guard column (12.5 mm × 4.6 mm × 5 *μ*m), with a flow rate of 2 mL/min, at a column temperature of 30°C and an injection volume of 5 *μ*L. The solvent used was a mixture of 75% acetonitrile and 25% water. The detector was a refractive index type while the data system was Agilent EZChrom Elite version 3.3. (Santa Clara, California, United States).

The pH and total titratable acidity (TTA) were measured according to Pearson's (1976). A quantity of 10 g of flour sample was suspended in 50 mL of deionized water for 5 min and pH measured, using a digital pH meter. The flour sample was treated as above and 10 Ml aliquot was titrated with 0.1 mol\L NaOH. The titre value was recorded. The TTA value was then calculated as the citric acid equivalent as follows: 1 mL NaOH = 0.009 mg citric acid.

Color properties were measured using a Minolta Chromameter (Collado et al. 1997). Thereafter , 10 g of flour was placed in a 1 cm high cylindrical Petri dish and measurement of the color was taken on the flattened surface of the flour. The chromometer was calibrated before the measurements, using the white calibration plate provided. Hunter L* values range from 100 (white) to 0 (black), a* values range from +a (green) to –a (red), and b* values range from +b (yellow) to –b (blue).

Minerals (Na, K, Mg, Ca, Fe, Zn, Cu, and Mn) were determined using an Atomic Absorption Spectrophotometer (James 1995). Phosphorus was determined colorimetrically using ammonium vanadate reaction (James 1995).

#### Functional properties of flour

Water absorption capacity was determined by the method of Sosulki et al. (1976) as described by Akubor (1997). Then 2 g of flour sample was mixed with 20 mL distilled water and allowed to stand at room temperature (30 ± 2°C) for 30 min, then centrifuged for 30 min at 2000 rpm (537 g). The volume of decanted supernatant fluid was measured and volume of water retained/bound per g of sample calculated. WAC was expressed as g of water bound/100 g of flour.

Swelling power and water solubility of the flour were estimated as described by Aina et al. (2009). A flour–water slurry (0.35 flour in 12.5 mL of distilled water) was heated in a water bath at 60°C for 30 min, with constant stirring. The slurry was centrifuged at 3000 rpm (1207 g) for 15 min, the supernatant was decanted into a weighed evaporating dish and dried at 100°C to constant weight. The difference in weight of the evaporating dish was used to calculate the water solubility. Swelling power was obtained by weighing the residue after centrifugation and dividing by original weight of the flour on dry weight basis.

Pasting profile was determined using a Rapid Visco Analyzer (RVA‐4; Newport Scientific Pty. Ltd., Australia) as described by Shittu et al. (2007) and also by following the instructions of the manufacturer. The RVA was interfaced with a personal computer equipped with the Thermocline for Windows software provided by the same manufacturer. 3 g of the sample (14% moisture basis) and 25 mL of distilled water was used. The equivalent sample mass (S) and water mass (W) corrected for 14% moisture basis was calculated using the formulae: *S* = 86 × *A*/100−*M*,* W* = 25 + (*A*−*S*), where *S *= corrected sample mass, *A *= sample weight at 14% moisture basis, *M *= actual moisture content of the sample (% as is), *W *= corrected water mass. A programed heating and cooling cycle was used at constant shear rate, where the slurry was held at 50 °C for 1 min, heated to 95°C within 7.5 min, and then held at 95°C for 5 min. It was subsequently cooled to 50°C within 8.5 min and held at 50°C for 2 min, while maintaining a rotation speed of 160 rpm. Total cycle time was 23 min. Duplicate tests were performed in each case. The viscosity is expressed as rapid visco units (RVU). The parameters measured automatically by the RVA were: peak viscosity (the highest viscosity of the paste during the heating phase), trough (lowest viscosity of the paste during the heating phase), breakdown viscosity (the difference between the peak viscosity and the trough), setback viscosity (the difference between the final viscosity and the trough), final viscosity (the viscosity at the end of the cycle), pasting temperature (ºC) (the temperature at which there is a sharp increase in viscosity of flour suspension after the commencement of heating), and peak time (min) (time taken for the paste to reach the peak viscosity).

### Data analyses

Descriptive analysis was performed to explore the general trend of the data. Multivariate analysis of variance was performed to compare the means of the samples for the several response variables. Significant difference was established at *P* < 0.05. Duncan's Multiple Range test was performed to separate the means where a significant difference exists. A multivariate General Linear Model (GLM) analysis was performed to determine the individual and interactive effects of the treatments (variety, pretreatment and drying methods) on the attributes measured. Significant effects were established at *P* < 0.05, 0.01 and 0.001 levels. Pearson's correlation coefficient among the quality attributes was calculated. Statistical packages used were Microsoft Excel and SPSS Version 17.0 (SPSS Inc., Chicago, IL).

## Results and Discussion

### Chemical properties of sweet potato flour

All the sweet potato (SP) flour differ significantly (*P* ≤ 0.05) in all the chemical properties investigated. Table 2 shows the range of values for proximate composition of flour as affected by variety, pretreatment, and drying methods, with moisture content (8.06–12.86%), protein (0.55–5.87%), fat (0.04–1.45%), fiber (0.08–5.54%), ash (0.15–2.09%) and carbohydrate (74.55–90.92%). Moisture content of the flour is within the range of 2.50–13.2% reported for sweet potato flour (van Hal 2000; Osundahunsi et al. 2003; Aina et al. 2009). Local white‐fleshed (LWF), Local yellow‐fleshed (LYF) and CIP Tanzania had the highest moisture contents (10.84–12.86%). A value of 12.5% has been considered as critical moisture content of flour within a locality with an ambient temperature of 27–29°C while a value of 10% has been recommended for long term storage (van Hal 2000). Moisture content of SP flour is considered a quality characteristic where storage is concerned, since water can accelerate chemical or microbiological deterioration (van Hal 2000). Since moisture content is directly related not only to drying method, but also conditions of temperature and time (Falade and Solademi 2010), the moisture content could be controlled to a desired level bearing in mind other quality requirements other than storage alone. In terms of pretreatment, the order of moisture content range for the flour was: potassium metabisulphite (8.06–12.74 ± 1.16) < water (8.36–12.80 ± 1.29) < blanched (8.97–12.80 ± 1.03) < untreated (9.35–12.86 ± 1.09). Sun–dried flour had lower moisture range (8.36–12.78 ± 1.26) than oven‐dried flour (8.54–12.86 ± 1.12).

**Table 2:** Range of values for proximate composition of sweet potato flour (n = 80) as affected by variety, pretreatment and drying method

	Moisture (%)	Protein (%)	Fat(%)	Fiber (%)	Ash (%)	Carbohydrate (%)
Minimum	8.06	0.55	0.04	0.08	0.15	74.55
Maximum	12.86	5.87	1.45	5.54	2.09	90.92
Mean	10.79	2.42	0.49	1.70	1.51	83.13
SD	1.13	1.28	0.42	1.43	0.61	2.85
CV (%)	10.44	52.66	86.96	84.17	40.51	3.43
Main effects						
Variety (V)	***	***	***	***	***	***
Pretreatment (P)	NS	***	NS	***	***	***
Drying (D)	***	***	*	NS	***	***
Interactive effects						
V × P	***	***	***	***	***	***
V × D	***	***	***	NS	***	***
P × D	***	***	NS	NS	***	***
V × P × D	***	***	*	NS	***	***

CV, Coefficient of variation.

*, **, *** Indicate significant effects at *P* < 0.05, 0.01, 0.001, respectively; NS indicate not significant.

In SP flour, carbohydrates account for the bulk of the flour and hence serve as a good energy source (Woolfe 1992). Flour from the two local varieties and TIS 8250 had the highest carbohydrate values (84.50–90.92%). Flour from 199034.1 could be targeted for low calorie, high nutrient foods because they had the lowest carbohydrate values (74.55–79.12%) but had the highest protein (3.77–5.87%), crude fiber (3.85–5.54%) and ash (1.68–2.02%) contents. Flour from the local varieties was generally high in carbohydrate but low in protein, fat, fiber, and ash. Protein is essential in the human diet for growth. Although SP is regarded as a high‐energy, low‐protein food, sweet potato protein in both fresh and flour form has been reported to be of good biological value (van Hal 2000; International Life Sciences Institute (ILSI) 2008). Hence, it could serve as a fairly important protein source among low‐income consumers in developing countries whose diets contain protein derived mostly from foods of vegetable origin. Ash content is a reflection of the mineral content of a food material. Lower values of protein, crude fiber and ash observed with flour from some varieties used in this study may be due to varietal differences (Woolfe 1992; van Hal 2000). It may also be due to the effect of some pretreatments as Jangchud et al. (2003) and Osundahunsi et al. (2003) reported that pretreatments involving leaching such as blanching and parboiling decreased the protein, crude fiber, and ash contents of SP flour.

The range of values for starch, sugar profile, and total titratable acidity (TTA) of SP flour as influenced by variety, pretreatment and drying method are shown in Table 3. The range of values of these components were starch (55.76–83.65%), amylose (10.06–21.26%), fructose (0.46–29.41 *μ*g/mg), glucose (1.19–32.14 *μ*g/mg), sucrose (4.90–113.18 *μ*g/mg), total sugar (22.39–125.46 *μ*g/mg) and TTA (0.02–0.13%). Although flour from the two local varieties of SP had the least starch content, however, they contained the highest amylose and medium range of total sugar. Flour from TIS 87/0087 and 2532 OP‐1‐13 had the highest starch while TIS 8250 flour had the least total sugar. In terms of pretreatment, the order of starch content of the flour was: water (55.76–73.18 ± 5.55%) < potassium metabisulphite (59.76–78.81 ± 6.02%) < untreated (60.17–79.55 ± 5.95%) < blanched (60.21–83.65 ± 6.68%). Blanched flour had the lowest ranges of fructose (0.46–12.75 ± 3.59%), glucose (1.19–12.47 ± 3.48%), sucrose (12.74–73.54 ± 17.53%), and total sugar (22.39–96.17 ± 20.36%). The range of total sugar for the flour was in the order: blanched (22.39–96.17 ± 20.36 *μ*g/mg) < water (46.29–111.49 ± 18.31 *μ*g/mg) < untreated (48.29–119.82 ± 18.57 *μ*g/mg) < potassium metabisulphite (54.55–125.46 ± 21.34 *μ*g/mg). Sun‐dried flour had slightly lower starch content (55.76–78.45 ± 6.69%) than oven‐dried flour (56.38–79.55 ± 6.67%). Sun‐dried flour also had lower sucrose (4.09–110.67 ± 21.75 *μ*g/mg) and total sugar (46.29–124.47 ± 19.96 *μ*g/mg).

**Table 3:** Range of values for chemical composition of sweet potato flour (n = 80) as affected by variety, pretreatment and drying method

	Starch (%)	Amylose (%)	Fructose (*μ*g/mg)	Glucose (*μ*g/mg)	Fructose (*μ*g/mg)	Total sugar (*μ*g/mg)	TTA (%)
Minimum	55.76	10.06	0.46	1.19	4.90	22.39	0.02
Maximum	83.65	21.26	29.41	32.14	113.18	125.46	0.13
**Mean**	69.05	15.97	5.90	7.31	57.66	70.87	0.07
**SD**	6.82	3.92	5.01	5.82	23.01	24.68	0.03
**CV(%)**	9.87	24.54	84.84	79.64	39.91	34.82	36.32
Main effects							
Variety (V)	***	***	***	***	***	***	***
Pretreatment (P)	***	***	***	***	***	***	***
Drying (D)	***	***	***	***	***	***	*
Interactive effects							
V × P	***	***	***	***	***	***	***
V × D	***	***	***	***	***	***	***
P × D	***	***	***	***	***	**	**
V × P × D	***	***	***	***	***	***	***

CV, Coefficient of variation; NS, indicates not significant.

*, **, *** indicate significant effects at *P* < 0.05, 0.01, 0.001, respectively.

The starch content of the flour (56–84%) was similar to the range of values (57–85%) reported by van Hal (2000). Starch is the predominant fraction of the dry matter of SP tubers (Ravindran et al. 1995). It is a rich source of carbohydrate and hence energy in the diet. It is a dominant factor that determines the physicochemical, rheological, and textural properties of starch‐based food products. It is also an important source of industrial raw materials for products such as noodles and glucose syrup, hence TIS 87/0087 and 2532 OP‐1‐13 flour with the highest starch content could be useful in these respect.

Amylose content of the flour (10.06–21.26%) was lower than the range of values (15.3–31.2%) reported by Aina et al. (2009) for flour from twenty‐one Caribbean SP cultivars. Report of amylose content of SP flour in literature is limited; however values obtained in this study are similar to those reported for SP starches; 19.1% (Collado et al., 1999) and 13.9–21.1% (Noda et al., 1995). Osundahunsi et al. (2003) and Garcia and Walter (1998) however reported higher values of 20.48–25.54%, and 32–34%, respectively, for SP starches. Osundahunsi et al. (2003) suggested that the high values reported in their study may be due to the highly sensitive Differential Scanning Calorimetry method used. Flour from LWF and LYF had the highest amylose content. The starch and amylose composition of staple food materials determines the processing and consumption characteristics of food products (Shittu et al. 2007).

The higher variability in fructose and glucose exhibited by the flour compared to the sucrose and total sugar suggests that fructose or glucose could be used to classify the flour into distinct groups. Sucrose is the dominant sugar contributing to the total sugar in SP flour, hence a good indicator to estimate one another. For staple foods in Nigeria, which are usually characterized by a bland taste, TIS 8250 flour with the least total sugar would be preferred. Flour from EX‐OYUNGA had the highest total sugar content and hence could be suitable to provide natural sweetness in the manufacture of food products. Generally, among the varieties, except for EX‐OYUNGA, blanched flour had the lowest starch and sugar content. This suggests that during blanching which takes place at an elevated temperature, the soluble sugars and starch in the sweet potato slices leached into the water, resulting in a reduction in the starch and sugar content of the flour. The result in this study is in agreement with that of Jangchud et al. (2003) which reported that blanching caused a reduction in starch; however it differs from the same report with respect to an increase in reducing sugar content of SP flour. The order observed for range of total sugar among the flour with respect to flesh color of the fresh roots is: white‐fleshed < yellow‐fleshed < orange‐fleshed. Significant (*P* < 0.01) positive correlations between total sugar and two tristimulus color parameters a* (0.416) and b*(0.588) were obtained.

Table 4 shows the mineral composition of the SP flour. The range values of the minerals were: Na (0.06–0.18%), K (0.76–1.22%), Mg (0.04–0.15%), Ca (0.09–0.29%), P (0.07–0.19%), Fe (20.65–45.35 mg/kg), Zn (18.85–33.75 mg/kg), Cu (3.60–8.50 mg/kg) and Mn (8.80–16.55 mg/kg). Flour from LWF and TIS 8250 were high in Fe, with LWF containing the highest. Arrowtip flour contained the highest Cu and Mn, while TIS 8250 contained the highest Zn. LYF flour was not only low in Fe, but they also contained the least Zn and Cu. The least Mn content was found among the 199034.1 flour. Generally, for all the pretreatments, the ranges of minerals in the flour were: (0.04–1.22%) for Na, K, Mg, Ca and P, (20.65–45.35 mg/kg) for Fe, (18.85–33.75 mg/kg) for Zn, (3.60–8.50 mg/kg) for Cu, and (8.80–16.55 mg/kg) for Mn. For oven‐dried flour the values were: (0.05–1.22%) for Na, K, Mg, Ca and P, (20.65–45.35 mg/kg) for Fe, (18.35–33.75 mg/kg) for Zn, (3.60–8.50 mg/kg) for Cu, and (9.10–16.55 mg/kg) for Mn. Sun‐dried flour however contained (0.06–0.25%) of Na, K, Mg, Ca and P, (21.45–42.45 mg/kg) of Fe, (19.70–31.60 mg/kg) of Zn, (4.15–7.85 mg/kg) for Cu, and (8.80–15.40 mg/kg) for Mn.

**Table 4:** Range of values for mineral composition of sweet potato flour (n = 80) as affected by variety, pretreatment and drying method

	Na (%)	K (%)	Mg (%)	Ca (%)	P (%)	Fe (mg/kg)	Zn (mg/kg)	Cu (mg/kg)	Mn (mg/kg)
Minimum	0.06	0.76	0.04	0.09	0.07	20.65	18.85	3.60	8.80
Maximum	0.18	1.22	0.15	0.29	0.19	45.35	33.75	8.50	16.55
Mean	0.10	0.94	0.09	0.19	0.13	33.13	25.78	5.54	12.73
SD	0.03	0.10	0.03	0.04	0.03	7.23	3.65	1.05	1.68
CV(%)	28.94	10.59	29.67	23.12	22.68	21.83	14.14	18.97	13.19
Main effects									
Variety (V)	***	***	***	***	***	***	***	***	***
Pretreatment (P)	***	***	*	***	NS	***	***	***	***
Drying (D)	**	***	NS	***	NS	**	***	NS	***
Interactive effects									
V × P	***	***	***	***	***	***	***	***	***
V × D	***	***	***	***	***	***	***	***	***
P × D	NS	*	***	***	***	***	***	**	***
V × P × D	***	***	***	***	***	***	***	***	***

CV: Coefficient of variation; NS, indicates not significant.

*, **, *** indicate significant effects at *P* < 0.05, 0.01, 0.001, respectively.

The individual mineral composition of SP flour is rarely studied; rather the ash content which is an estimate of the total mineral content is usually reported. Potassium was the major mineral present in the SP flour confirming the report by Ravindran et al. (1995). The results in this study showed that SP roots are moderately good sources of minerals particularly essential micronutrients such as Fe, Cu, Zn, and Mn. According to van Hal (2000), SP flour is estimated to contribute 20–40% of the Recommended Daily Allowance of Fe.

### Main and interactive effects of variety, pretreatment and drying methods on chemical properties of sweet potato flour

The interactive effects of variety, pretreatment and drying methods on the chemical properties of SP flour are presented in Tables 2, 3, 4. The factors that had the most significant (*P* < 0.001) effect on all the chemical properties of the SP flour were variety and the interaction between variety and pretreatment. Protein, ash, and carbohydrate were significantly (0.001) affected by each of the factors and all the interactions (Table 2). Pretreatment had no significant effect (*P* > 0.05) on moisture and fat content of the flour. Starch, amylose and all the sugars were significantly (*P* < 0.001) affected by each of variety, pretreatment, drying method as well as all the interactions among these factors (Table 3). Ca, Zn and Mn were significantly (*P* < 0.001) affected by each of the factors and all the interactions (Table 4). Drying did not have a significant (*P* ≥ 0.05) effect on Mg, P and Cu.

### Functional properties of sweet potato flour

Table 5 shows the Hunter L* a* b* color values of SP flour as affected by variety, pretreatment and drying methods. The range of values for all the flour were: L* (79.90–101.48 ± 5.44), a* (−0.27–3.54 ± 0.82) and b* (9.89–27.94 ± 4.02). In terms of variety, the order of mean L* values was white‐fleshed (87.79 ± 4.76) < orange‐fleshed (95.17 ± 2.03) < yellow‐fleshed (95.91 ± 2.66), while that of mean a* values was white‐fleshed (14.22 ± 2.64) < yellow‐fleshed (15.79 ± 1.47) < orange‐fleshed (21.92 ± 4.23). The mean L*: a*: b* values showed considerable difference between flour made from the two orange‐fleshed varieties; Ex‐oyunga (96.15: 2.49: 25.60) and 199034.1 (94.18: 0.39: 18.25). In terms of pretreatment, the order of mean L* values was: potassium metabisulphite (91.48 ± 5.19) < untreated (91.58 ± 5.23) < water (91.71 ± 6.54) < blanched (92.05 ± 5.07), and b* values was: blanched (14.56 ± 3.99) < potassium metabisulphite (16.71 ± 4.19) < water (16.73 ± 3.74) < untreated (16.92 ± 3.95). Sun‐dried flour had higher mean values of L* (91.75 ± 5.58) and a* (0.96 ± 0.75) than oven‐dried flour (L* 91.66 ± 5.37, a* 0.73 ± 0.88), however, oven‐dried flour had higher b* values (16.61 ± 4.33) than sun‐dried flour (15.85 ± 3.69).

**Table 5:** Range of values for functional properties of sweet potato flour (n = 80) as affected by variety, pretreatment and drying method

	L*	A*	B*	WAC	WS	SWP
Minimum	79.90	−0.27	9.89	140.00	6.89	1.66
Maximum	101.48	3.54	27.94	280.00	26.18	5.00
Mean	91.70	0.84	16.23	194.13	15.30	3.31
SD	5.44	0.82	4.02	25.97	3.80	0.50
CV(%)	5.93	97.21	24.74	13.38	24.80	14.97
Main effects						
Variety (V)	***	***	***	***	***	***
Pretreatment (P)	NS	***	***	***	***	***
Drying (D)	NS	***	***	***	***	*
Interactive effects						
V × P	***	***	***	***	***	***
V × D	***	***	***	***	***	***
P × D	***	***	NS	*	***	***
V × P × D	***	***	***	***	***	***

CV, Coefficient of variation; WAC, Water absorption capacity; WS, Water solubility; SWP, Swelling power; L*,Lightness; a*,Redness; b*, Yellowness.

*, **, *** indicate significant effects at *P* < 0.05, 0.01, 0.001, respectively; NS indicates not significant.

The tristimulus color parameter L* indicate whiteness of the flour, as reported by Collado et al. (1997). In the present study however, flour from white‐fleshed varieties had the least mean L* values (87.79). This confirms the works of other authors (van Hal 2000) which have shown that the whiteness of the flour is not always directly related to the flesh color of the roots. They suggested that this is an indication of the high level of browning that occurs during drying of SP chips and processing the flour. The b* parameter may be a better measure of the color and intensity of flour from colored varieties, this is because flour from the two orange‐fleshed varieties, 199034.1 and EX‐OYUNGA, had the highest b* values (17.15–27.94) which increased with intensity. Flour from these orange‐fleshed varieties could add natural color to food products. Flour from the white varieties, LWF and TIS 87/0087, would be suitable where whiteness is desired because they have the least b* values (10.25–13.66). Flour produced by blanching showed the least mean b* values (14.56 ± 3.99), this is supported by the report of Jangchud et al. (2003). Aina et al. (2009) reported that flour from the orange‐fleshed varieties were characterized by higher a* and b* values, lower amylose, but higher total sugar levels. In the present study however, only flour from EX‐OYUNGA (an intensely‐colored orange‐fleshed variety) agrees with this description, flour from 199034.1, a less intensely‐colored orange‐fleshed variety did not show these characteristics.

The water absorption capacity (WAC), water solubility (WS), and swelling power (SWP) of SP flour as affected by variety, pretreatment, and drying methods is shown in Table 5. The ranges of values for all the flour were: WAC (140–280 ± 25.97), WS (6.89–26.18 ± 3.80) and SWP (1.66–5.00 ± 0.50). In terms of variety, flour from EX‐OYUNGA had the highest WAC (215–280) and WS (18.30–26.18) while LYF had the highest SWP (3.05–5.00) but the least WAC (140–170). Flour from TIS 8250 and SHABA had the least WS (7.49–16.83), while 2532 OP‐1‐13 flour had the least SWP (2.69–2.99). Generally, blanched flour had the least mean values of WAC, WS and SWP while potassium metabisulphite‐treated flour had the highest mean values of WAC and SWP. Untreated flour had the highest WS. Oven‐dried flour had higher mean values of WAC (197.50 ± 26.94), WS (16.18 ± 3.45) and SWP (3.34 ± 0.55) than sun‐dried flour with WAC (190.75 ± 24.85), WS (14.43 ± 3.97) and SWP (3.29 ± 0.44).

The functional properties of flour are those that directly determine their end uses. The WAC of SP flour in this study is much higher than reported values (24–42) by Osundahunsi et al. (2003) for native and parboiled flour. The authors noted that precious studies reported higher values and suggested that the varieties tested have unusually low WAC. Water absorption capacity is the ability of the starch or flour to absorb water and swell for improved consistency. It is desirable in food systems to improve yield and consistency and give body to food. Osundahunsi et al. (2003) reported that parboiling improved the WAC of the flour by 173–175% and suggested that it should be used for SP flour required as a thickener. Jangchud et al. (2003) also reported that blanching increased the SWP of flour at all temperatures investigated. In the present study however, blanched flour had the least mean value of WAC (191.67) while WS and SWP compared to native flour. The relatively lower values of WAC could be due to some genetic factors rather than the pretreatment.

### Main and interactive effects of variety, pretreatment and drying methods on functional properties of sweet potato flour

Each of the Hunter color parameters (L*, a*, b*) were significantly (*P* < 0.001) affected by variety, as well as the combinations of variety and pretreatment, variety and drying, and variety, pretreatment, and drying (Table 5). The a* values were significantly (*P* < 0.001) affected by each of the main factors and all their combinations. The WAC, WS, and SWP were all significantly (*P* < 0.001) affected by variety, pretreatment, and the combinations of variety and pretreatment, variety and drying, as well as variety, pretreatment, and drying. WS was significantly affected by all the main factors and their interactions (Table 5). Drying, and the interaction between pretreatment and drying, affected SWP and WAC, respectively, only at *P* < 0.05. The implication of these interactive effects is that each of the main factors as well as the combinations are very important to the functional properties of flour and should therefore be selected appropriately during processing of SP flour.

### Pasting properties of sweet potato flour

Table 6 shows the pasting properties of SP flour as affected by variety, pretreatment, and drying methods. The range of values were peak viscosity (PV) (24.50–260.92 RVU), trough (T) (7.08–145.83 RVU), breakdown viscosity (BV) (11.00–125.33 RVU), final viscosity (FV) (10.21–225.1), setback viscosity (SBV) (3.04–92.21 RVU), peak time (PTm) (6.07–9.76 min), and peak temperature (PTp) (69.78–81.25°C). Sun‐dried flour had higher ranges for almost all the pasting properties PV (47.88–260.92 ± 53.12), T (17.21–145.83 ± 31.54), BV (13.25–125.33 ± 29.96), FV (26.04–222.50 ± 55.95), SBV (7.50–92.21 ± 25.82) than oven‐dried flour PV (24.50–197.00 ± 47.02), T (7.08–108.58 ± 33.63), BV (11.00–103.79 ± 25.70), FV (10.21–200.21 ± 59.14), and SBV (3.04–91.83 ± 26.33).

**Table 6:** Range of values for pasting properties of sweet potato flour (n = 80) as affected by variety, pretreatment and drying method

	Peak viscosity (RVU)	Trough (RVU)	Breakdown viscosity (RVU)	Final viscosity (RVU)	Setback viscosity (RVU)	Peak time (min)	Pasting temperature (^0^C)
Minimum	24.50	7.08	11.00	10.21	3.04	6.07	69.78
Maximum	260.92	145.83	125.33	225.50	92.21	9.76	81.25
Mean	136.51	77.67	57.18	125.15	46.65	6.86	78.27
SD	52.61	34.48	25.72	60.55	27.13	0.52	2.96
CV (%)	38.54	44.40	44.98	48.38	58.16	7.51	3.78
Main effects							
Variety (V)	***	***	***	***	***	***	***
Pretreatment (P)	***	***	***	***	***	***	***
Drying (D)	***	***	***	***	***	***	***
Interactive effects							
V × P	***	***	***	***	***	***	***
V × D	***	***	***	***	***	***	***
P × D	***	***	***	***	***	***	***
V × P × D	***	***	***	***	***	***	***

CV, Coefficient of variation; NS, indicates not significant.

*, **, *** Indicate significant effects at *P* < 0.05, 0.01, 0.001, respectively.

The most common objective method of determining the cooking property of starch‐based food products is through an amylograph pasting profile. Such information have been used to correlate the functionality of starchy food ingredients in processes such as baking (Defloor et al. 1995; Rojas et al. 1999) and extrusion cooking (Ruales et al. 1993). In these studies, it was observed that the pasting properties (such as peak, trough, setback, breakdown, and final viscosities) of the cooked flour showed the widest variation amongst other pasting properties measured. This agrees with the results in this study in which the pasting viscosities varied between 39% and 58%, while the pasting time and temperature varied between 4–8% (Table 6). The values obtained for all the pasting properties except pasting time were much higher than those reported by Jangchud et al. (2003). The highest Peak viscosity (PV), Breakdown viscosity (BDV), and Final viscosity (FV) were found among the TIS 8250 flour in addition to having high *T* values. Flours from EX‐OYUNGA had very low pasting viscosities, an indication of high enzymic activities in the flour (Osundahunsi et al. 2003; Collado and Corke, 1999).

The pasting temperature of the flour were higher than values reported by Osundahunsi et al. (2003) but lower than those reported by Aina et al. (2009) and Jangchud et al. (2003). Pasting temperature of flour can have energy‐cost implications. It had earlier been reported that high pasting temperatures of flour may be due to higher amylose contents of the varieties (Aina et al. 2009). However, in this present study, there was a negative significant (*P* < 0.001) correlation between amylose content and pasting temperature, indicating that sweet potato flour with a higher amylose content is expected to have lower pasting temperature. Aina et al. (2009) also suggested that sugar content of the flour may have influenced the pasting temperature by competing with the starches for moisture, decreasing swelling, and viscosity while increasing the pasting temperature. The present study agrees with the low pasting viscosities due to high sugar, however the significant (*P* < 0.01) but low (*r* = 0.322–0.395) correlation shows that a high swelling power is expected. There was no significant (*P* > 0.05) correlation between sugars and pasting temperature. The setback viscosity indicates the retrogradation tendency of starch and flour. Low setback values showed a lower tendency for retrogradation. According to Garcia and Walter (1998), cultivars with the highest amount of amylose are expected to have stiffer pastes and high setback viscosities. There was no significant (*P* > 0.05) correlation between amylose and setback viscosities of flour in this study. Nevertheless, LYF flour which had the highest amylose content showed relatively high setback viscosities. Flour from 2532 OP‐1‐13 and EX‐OYUNGA had the least setback values (3.04–43.42 RVU), hence suitable for pie fillings where retrogradation may cause syneresis and low quality product. Nevertheless, for all the varieties, except 2532 OP‐1‐13 and 199034.1, blanching resulted in a considerable decrease in values of all the pasting properties. This confirms the report of Jangchud et al. (2003) in which peak, final, and breakdown viscosities were lower than those of unblanched flour. In the same report, blanching significantly increased the pasting temperature by about 6–20°C for the two varieties studied. In the present study however, a difference of 1–2°C was observed which may be an increase or decrease depending on variety and the interaction between pretreatment and drying method.

### Main and interactive effects of variety, pretreatment, and drying methods on pasting properties of sweet potato flour

In this study, no particular trend was observed in pasting properties as a result of the treatments. This could imply that although the pasting properties were significantly (*P* < 0.001) affected by variety, pretreatment, and drying methods as well as the interactions among these treatments (Table 6), genetic variation may be a more dominant factor influencing the pasting properties of the SP flour.

### Correlations among the properties of sweet potato flour

Table 7 shows the linear correlations between chemical composition and functional properties of sweet potato flour. Significant (*P* < 0.01) negative correlations were observed between L* values and carbohydrate, starch, amylose, and glucose while the significant (*P* < 0.01) correlations between b* values and protein, fat, fiber, ash, and sugars were positive. WAC was negatively correlated with moisture content, but showed positive correlations with protein, fat, fiber, and ash (*P* < 0.01). The WS showed positive correlations (*P* < 0.01) with all the sugars particularly sucrose (*r* = 0.801) and total sugar (*r* = 0.882). The SWP showed significant (*P* < 0.01) negative correlations with starch but positive correlations with sucrose and total sugar. Generally, the lower the moisture contents of the flour, the higher the WAC. On the other hand, the higher the sugar contents, the higher the WS of the flour. Furthermore, lower starch content favors high SWP.

**Table 7:** Correlations between chemical composition and functional properties of sweet potato flour

Chemical property	L*	a*	b*	WAC	WS	SWP
Moisture content	−.041	−.272**	−.328**	−.402**	−.120	.095
Protein	.380**	.168*	.426**	.458**	−.100	.051
Fat	.072	.249**	.517**	.431**	.391**	.118
Fiber	.134	.147	.478**	.493**	.226**	.241**
Ash	.157*	.206**	.291**	.341**	.051	−.255**
Carbohydrate	−.263**	−.108	−.423**	−.410**	−.084	−.150
Starch	−.241**	−.029	−.079	−.084	.109	−.420**
Amylose	−.218**	−.083	−.143	−.210**	.067	.028
Fructose	−.066	.514**	.323**	.068	.327**	−.059
Glucose	−.234**	.372**	.191*	.176*	.292**	−.144
Sucrose	.090	.241**	.512**	.167*	.801**	.395**
Total `sugar	.016	.416**	.588**	.211**	.882**	.322**
TTA	−.124	.323**	.357**	.380**	.455**	.045

L*, Lightness; a*, Redness; b*,Yellowness; WAC, Water absorption capacity; WS, Water solubility; SWP, Swelling power; TTA, Total Titratable acidity.

*Correlation is significant at *P* < 0.05.

**Correlation is significant at *P* < 0.01.

The pasting temperature showed significant (*P* < 0.01) correlations with more chemical properties than any other pasting property (Table 8). A strong positive correlation (*r* = 0.728) was particularly observed between pasting temperature and ash. Correlation showed that the lower the moisture content, the higher the pasting temperature. Fat showed negative but significant correlations (*P* < 0.01) with PV, T, BDV, FV, and SBV. Sucrose and total sugar showed similar trends as fat, except with SBV which was significant for sucrose only at *P* < 0.05. The lower the WAC and WS, the higher the peak, trough, breakdown, final, and setback viscosities as indicated by the significant negative correlations between these functional and pasting properties (Table 9). Peak time and peak temperature were positively correlated with WAC and this was significant (*P* < 0.01). SWP showed a significant negative correlation (*P* < 0.01) with breakdown viscosity and pasting temperature.

**Table 8:** Correlations between chemical and pasting properties of sweet potato flour

Chemical property	Peak viscosity (RVU)	Trough (RVU)	Break down viscosity (RVU)	Final viscosity (RVU)	Set back viscosity (RVU)	Peak time (min)	Pasting temperature (^0^C)
Moisture content	.284**	.326**	.049	.337**	.292**	.038	−.401**
Protein	.072	.191*	−.026	.165*	.166*	.351**	.467**
Fat	−.375**	−.340**	−.260**	−.362**	−.352**	−.032	.240**
Fiber	−.268**	−.136	−.311**	−.173*	−.187*	.231**	.337**
Ash	−.047	−.062	.122	−.067	−.007	.418**	.728**
Carbohydrate	.042	−.099	.158*	−.069	−.057	−.375**	−.390**
Starch	−.111	−.224**	.157*	−.190*	−.099	.161*	.457**
Amylose	.077	.049	.027	.060	.041	−.244**	−.360**
Fructose	−.008	−.135	.196*	−.102	−.042	−.112	.028
Glucose	−.115	−.298**	.209**	−.297**	−.263**	−.182*	.056
Sucrose	−.334**	−.328**	−.240**	−.269**	−.183*	−.047	.065
Total `sugar	−.340**	−.403**	−.136	−.342**	−.241**	−.109	.080
TTA	.048	−.011	.210**	.008	.079	.185*	.385**

TTA, Total Titratable Acidity.

*Correlation is significant at *P* < 0.05.

**Correlation is significant at *P* < 0.01.

**Table 9:** Correlations between functional and pasting properties of sweet potato flour

Functional property	Peak viscosity (RVU)	Trough (RVU)	Breakdown viscosity (RVU)	Final viscosity (RVU)	Setback viscosity (RVU)	Peak time (min)	Pasting temperature (^0^C)
WAC	−.319**	−.206**	−.270**	−.266**	−.282**	.240**	.490**
WS	−.473**	−.505**	−.290**	−.437**	−.332**	−.146	−.003
SWP	−.026	.072	−.217**	.069	.029	−.037	−.258**

WAC, Water absorption capacity; WS, Water solubility; SWP, Swelling power; RVO, Rapid visco units.

*Correlation is significant at *P* < 0.05.

**Correlation is significant at *P* < 0.01.

In order to determine attributes that could serve as quality indicators for sweet potato flour irrespective of variety, pretreatment and drying method, attributes that were significantly (*P* < 0.001) affected by the interaction among these treatments were selected. Correlations that were significant (*P* < 0.01) among the selected attributes were identified. The sugars show significant positive correlations with WAC, water solubility, and swelling power. Consequently, it may be deduced from these significant correlations that amylose, sucrose, or total sugar, and water absorption capacity could serve as a reliable quality indicators to predict the pasting properties of sweet potato flour and hence the functionality in specific food products.

### Practical applications

Sensory acceptability of products such as ‘cooked paste’ (‘amala’) require sweet potato flour characterized by lower values of total sugar, water absorption capacity, swelling power amongst other properties (Fetuga et al. 2014). The correlations in this study indicates that sweet potato varieties that will give flour with low values of sugar, WAC and swelling power should be targeted for consumer acceptable ‘amala’.

High paste viscosities are desirable in flour used as thickeners, whereas low paste viscosities are desirable for high‐calorie food formulations such as weaning and speciality foods (Wiessenborn et al. 1994). The correlations in this study indicates that sweet potato varieties that will give flour with lower values of WAC and water solubility (WS) and consequently higher paste viscosities will be appropriate for thickening agents. On the other hand, varieties that will produce flour with high WAC and WS and consequently low paste viscosities, should be targeted at weaning and speciality foods.

## Conclusions

This study has shown that variety had a significant effect on all the attributes of sweet potato flour (SPF). Pretreatment did not significantly affect moisture, fat, and lightness of SPF. The drying method did not significantly affect fiber and lightness of SPF. The interactive effect of variety, pretreatment, and the drying method had a significant effect on all the quality attributes of SPF except fat and fiber. The sweet potato flour had significantly different chemical, functional, and pasting properties. The flour showed a wider variability in pasting properties. Blanching resulted in considerable decrease in paste viscosities irrespective of variety and drying method. Varietal difference was a dominant factor in the differences observed in quality attributes of the flour studied, therefore, sweet potato varieties should be targeted at specific end uses. Sucrose or total sugar and water absorption capacity could serve as reliable quality indicators to predict the pasting properties of sweet potato flour and hence functionality in specific food products.

## Conflict of Interest

None declared.
